# Construction of a comprehensive observer-based scale assessing aging-related health and functioning in captive rhesus macaques

**DOI:** 10.18632/aging.102219

**Published:** 2019-09-09

**Authors:** Wei Deng, Guoying Guan, Chong Xiao, Guangjin Qu, Jing Xue, Chuan Qin, Hui Han, Yuhong Wang

**Affiliations:** 1 Key Laboratory of Human Disease Comparative Medicine, Chinese Ministry of Health, Beijing Key Laboratory for Animal Models of Emerging and Remerging Infectious Diseases, Institute of Laboratory Animal Science, Chinese Academy of Medical Sciences and Comparative Medicine Center, Peking Union Medical College, Beijing, China; 2 Department of Geriatrics, The First Affiliated Hospital of Harbin Medical University, Harbin, China

**Keywords:** scale, health and functioning, aging, rhesus macaques, translational model

## Abstract

Aging-related health and functioning are difficult to quantify in humans and nonhuman primates. We constructed an observer-based scale for daily application in assessing the aging-related health and functioning of rhesus macaques. Ten items referring to an aging appearance, musculoskeletal aging and aging-related eating behavior were selected through a panel consensus. The Aging-related Health and Functioning Scale (AHFS) was constructed based on these scored items form 57 healthy rhesus macaques. High reliability of the AHFS was shown based on Cronbach’s alpha coefficient (0.877). The structure of the AHFS was validated by three exploratory factors. The largest factor, whose four components were dietary uptake, iliac muscle mass, hair condition and fragility, and sex, explained 50.5% of the variation in aging-related health and functioning scores. The second factor, involving age, tooth loss and tooth wear, explained 15.5% of the variation. The lowest-ranking factor comprised only facial redness and accounted for 10% of the variation. A hierarchical cluster analysis validated the good applicability of the scale in distinct samples. From these scale-scored results, complicated aging phenomena observed in humans, including the sex-survival paradox and the calorie-related health-survival paradox, were both demonstrated in rhesus macaques. Therefore, the AHFS provides a valuable approach for aging-related research.

## INTRODUCTION

Humans are growing older as a result of improvements in public health, producing an era of accelerated aging. Preservation of healthy aging and maintenance of functional ability is increasingly the focus of public health care in older humans [1, 2]. To achieve this goal, an integrated model reflecting the complex and multifaceted aging-related health and functioning based on daily life is highly desired [3, 4]. Rhesus macaques are the most extensively studied nonhuman primate animals exhibiting parallel aging characteristics and a majority of the age-related diseases of humans [5, 6]. Despite these close genetic relationships and the high degree of similarity of aging phenotypes, the repeatability and reproducibility of experimental findings in rhesus macaques has not been satisfactory [3]. One potential reason is the high heterogeneity in individual health and functioning that accumulates from the young-adult age to advanced age [7]. Therefore, although a large variety of scales have been used to assess aging-related health and functioning in humans, a well-accepted and widely used scale is still lacking for the assessment of elderly humans [8, 9]. It is definitely more difficult to develop an aging-related health and functioning scale in rhesus macaques because they are incapable of self-rating or being interviewed.

Although numerous studies have indicated that cardiovascular aging, motor aging [10] and mental aging all show similar declines in macaques [6] and in humans, the aging-related traits and trajectories were different between nonhuman primates and humans. From a biological perspective, arterial stiffness, which is highly prevalent in humans, is rare in aged monkeys with the development of ectasia being common in animals fed a normal diet [11]. Moreover, aging-related biological changes can be distinguished only by advanced techniques and comprehensive examinations. From a psychosocial perspective, it has been difficult to develop an observation-based scale because of the much lower psychosocial performance with a narrow range between the best and the worst levels of performance. Using the cognitive domain of aging-related health as an example, the cognitive capability in adult monkeys was comparable to that in an 8–10 month human infant [11]. Therefore, the largest scaling range of cognitive capability in monkeys was comparable to that from an infant to a newborn in humans. With such a narrow range of variation, daily observation with the naked eye could not make any particular assessment with a high degree of reliability. That is, such confined psychosocial performance of animals attenuated the availability of distinguishable items based on daily observation. To resolve this dilemma, routine studies on aging-related neurological changes have required training of animals over a period of time. This long training period allows numerous external factors to bias learning capability and longitudinal adaptability, greatly attenuating the repeatability and reproducibility of experiments with these nonhuman primate models; however, the potential impacts of such biases have long been neglected.

In the present study, we attempted to develop a rapid, easily operationalized scale based on daily observation to assess aging-related health and functioning in a rhesus macaque group. With high respect to the multidimensional nature of health and functioning, we have incorporated the social domain of health into the present nonhuman primate “society” for better efficacy. We believe that the present scale is valuable not only for human aging research but also for improving the repeatability and reproducibility of studies with nonhuman primate models.

## RESULTS

### Screening of the aging-related items to assess overall health and daily functioning

Initially, 15 items referring to perceived aging, physical aging and social aging were selected according to the consensus of the panel. Preliminary scaling results based on daily observations resulted in ten items that could be clearly discriminated with acceptable intraobserver agreement. The scale containing 12 items with each point description is shown in Table 1. The lower the score was, the milder the aging process and the better the health and functioning of the animal. All screened items were graded on a three-point scale with the exception of tooth loss. An optimization-based development on “Age” and “Sex” was performed based on the comparison of the respective scales’ Cronbach’s alpha coefficient. The scale scoring by “chronological age” or “categorized age” as well as scoring “male with lower score” or “female with lower score” underwent optimization. The age-dependent category was divided according to the percentile of chronological age.

**Table 1:** Items and scoring system for the AHFS based on daily life in rhesus macaque groups.

**Age (Categorized)**
1=junior
2=adult
3=senior
Sex
1=female
2=male
**Appearance (Domain of Perceived Aging)**
Facial redness
1=light facial redness
2=moderate facial redness
3=dark facial redness
Hair condition
1=overall glossy hair/good
2= overall moderate hair/moderate
3= overall gloomy hair/poor
Hair fragility
1=broken with high force/low fragility
2= broken with moderate force/moderate fragility
3= broken with low force/high fragility
**Musculoskeletal condition (Domain of Physical Aging)**
Tooth loss
1=without tooth loss
2=with tooth loss
Tooth wear
1=mild tooth wear
2=moderate tooth wear
3=severe tooth wear
Iliac muscle mass
1= thick
2=moderate
3=thin
**Performance in dietary uptake (Domain of Social Aging)**
Volume of dietary uptake (per meal)
1=large
2=medium
3=small
Average speed of dietary uptake
1=fast
2=medium
3=slow
Size of cheek pouch
1=large
2=medium
3=small
Eating order during a meal/social rank
0=First (Male) /dominance
1=Prior /high
2=Interior /moderate
3=Posterior/low

*Note: A description of the scoring system (using the 3-point system as an example)

Score=1 indicates the least aging-characterized health and functioning

Score= 2 indicates an intermediate level of aging-characterized health and functioning

Score=3 indicates the most aging-characterized health and functioning

### The profile of aging-related health and functioning in the rhesus macaque group assessed by the AHFS

The fundamental profile of aging-related health and functioning scored by the AHFS in the present rhesus macaque group are shown in Table 2. From the table, we can see that the majority of animals were in the “Adult category”. Notably, all these scores were assessed based on daily life performance, which indicated the small time commitment and relative noninvasive merit. A very short period of anesthesia was required to score the tooth condition and iliac mass in the male animals.

**Table 2:** The item-dependent scores for health and functioning in a representative rhesus macaque group.

**Item**	**Mean±SD**	**Trait (score)**		
		**Number (percentage)**		
Age*	14.05 ± 2.87	Junior (1)	Adult (2)	Senior (3)
		n=11 (19.30%)	n=35 (61.40%)	n=11 (19.30%)
Sex	-	Female (1)	Male (2)	
		n=29 (50.88%)	n=28 (49.12%)	
Facial redness	1.71 ± 0.67	Light (1)	Moderate (2)	Dark (3)
		n=23 (40.35%)	n=27 (47.37%)	n=7 (12.28%)
Hair condition	1.50 ± 0.73	Good (1)	Moderate (2)	Poor (3)
		n=36 (63.16%)	n=13 (22.81%)	n=8 (14.04%)
Hair fragility	1.33 ± 0.57	Low (1)	Moderate (2)	High (3)
		n=41 (71.93%)	n=13 (22.81%)	n=3 (5.26%)
Tooth loss	1.28 ± 0.45	Without (1)	With (2)	
		n=41 (71.93%)	n=16 (28.07%)	
Tooth wear	1.79 ± 0.59	Mild (1)	Moderate (2)	Severe (3)
		n=17 (29.82%)	n=35 (61.40%)	n=5 (8.77%)
Iliac muscle mass	1.42 ± 0.65	Thick (1)	Moderate (2)	Thin (3)
		n=38 (66.67%)	n=14 (24.56%)	n=5 (8.77%)
Volume of dietary intake	1.46 ± 0.65	Large (1)	Moderate (2)	Small (3)
		n=36 (63.16%)	n=16 (28.07%)	n=5 (8.77%)
Speed of dietary intake	1.52 ± 0.68	Fast (1)	Moderate (2)	Slow (3)
		n=33 (57.89%)	n=18 (31.58%)	n=6 (10.53%)
Size of cheek pouch	1.45 ± 0.60	Large (1)	Moderate (2)	Small (3)
		n=34 (59.65%)	n=20 (35.09%)	n=3 (5.26%)
Order of dietary intake	0.98 ± 1.14	Predominance* (0)		
		n=28 (49.21%)		
		Prior (1)	Interior (2)	Posterior (3)
		n=11 (19.30%)	n=9 (15.97%)	n=9 (15.79%)

*Note: Predominance (score=0) indicated males who always took the first meal in a group. The 3-point scoring of dietary intake was used to rank the order the females’ dietary intake in a group.

### The interrelationship of the aging-related items in the scale

Using Pearson correlations, we further analyzed the interrelationship of the scored items on aging-related health and functioning in the AHFS (Table 3).

**Table 3:** The correlation coefficients between health and functioning scores for each item.

**Item**	**Overall score for health and functioning**	**Age**	**Sex**	**Facial redness**	**Hair Fragility**	**Hair condition**	**Tooth loss**	**Tooth wear**	**Iliac muscle mass**	**Volume of dietary uptake**	**Speed of dietary uptake**	**Size of cheek pouch**
Age	0.11											
Sex	0.57^****^	-0.36^**^										
Facial redness	0.42^***^	0.09	0.20									
Hair Fragility	0.69^****^	0.19	0.28^*^	0.08								
Hair condition	0.77^****^	0.20	0.41^**^	0.11	0.73^****^							
Tooth loss	0.27^*^	0.57^****^	-0.22	0.26^*^	0.26	0.11						
Tooth wear	0.60^****^	0.46^***^	-0.01	0.39^**^	0.46^****^	0.42^**^	0.43^****^					
Iliac muscle mass	0.80^****^	0.06	0.44^***^	0.27^*^	0.51^****^	0.71^****^	0.14	0.47^***^				
Volume of dietary uptake	0.86^****^	-0.02	0.61^****^	0.17	0.51^****^	0.63^****^	0.10	0.34^***^	0.67^****^			
Speed of dietary uptake	0.85^****^	-0.01	0.53^****^	0.25	0.52^****^	0.54^****^	0.09	0.41^**^	0.57^****^	0.81^****^		
Size of cheek pouch	0.82^****^	-0.09	0.54^****^	0.28^*^	0.44^***^	0.58^****^	0.05	0.28^*^	0.60^****^	0.87^****^	0.80^****^	
Order of dietary uptake	0.80^****^	-0.24	0.79^****^	0.25	0.37^**^	0.52^****^	-0.06	0.23	0.58^****^	0.77^****^	0.77^****^	0.72^****^

Note: ^****^P<0.0001; ^***^ *P*<0.001; ^**^ *P*<0.01; ^*^ *P*<0.05

**Figure 1:**
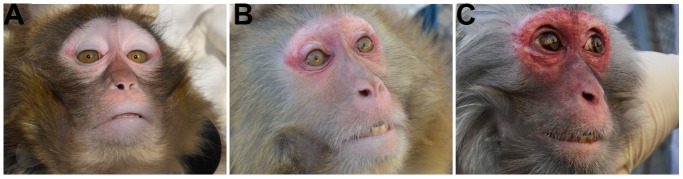
**Scored facial redness in the scale on aging-related health and functioning.** Perceived age was rated by darkness of facial redness on the present scale on aging-related health and functioning in rhesus macaques. Representative animals scored 1, 2 and 3 are shown in A, B and C, respectively. Reduced aging-related health and functioning was supposed to be in parallel to the increased darkness of facial redness. The high interrelationship of items in the scale indicated both convergent and discriminate validity.

On the present scale, the most interrelated domain was four items referring to dietary uptake. As the only “officially” social activity in the captive group, dietary uptake behaviors were supposed to parallel psychosocial behaviors during aging and were consistent with the findings in humans [14, 15]. More interestingly, the order of dietary intake displayed a considerably high interrelation with other items, further supporting our proposal of the great significance of social behaviors. The second most highly interrelated item was iliac muscle mass. The loss of muscles commonly occurred with variation in location and time of onset [16]. Therefore, we selected the iliac muscle, characterized as the largest and most easily palpated muscle, to grade the overall muscle condition. Hair fragility and hair condition also reached an approximately 0.7 correlation with overall scored health and functioning. We subjectively scored hair conditions and objectively measured hair fragility using an indirect method for increased accuracy. During the fragility measurement, we experienced a clear discrimination between fragile hair with easy breakage and tough hair with difficult breakage. Finally, facial redness displayed a mild and significant correlation with the scored health and functioning. Perceived age via facial appearance has been found to be an independent biomarker correlated with physical and cognitive function as well as survival in humans [17, 18]. As no method assessing perceptive age in nonhuman primates had been reported, we rated the darkness of facial redness, mirroring skin aging in the rhesus macaques, for greater sensitivity to perceived aging (Figure 1).

Reduced aging-related health and functioning was expected to be in parallel to the increased darkness of facial redness. The high interrelationship of items in the scale indicated both convergent and discriminant validity. The lowest correlation was shown for chronological age. Only dental condition was found to be correlated with chronological age. This finding suggested that chronological age was a poor determinant of the complexity of multidimensional nature of health and functioning.

### The reliability of the AHFS in rhesus macaques

The reliability was 0.877 (Cronbach’s alpha coefficient), indicating that the items in the scale measure as a latent construct with 87.75% internal consistency. Notably, during the development of the scale, we compared Cronbach’s alpha coefficient by denoting the male or female animals as milder aging (score=1). We found that when the female sex was rated with a milder aging score, higher reliability scores were obtained. Because higher socioeconomic status has been closely correlated with better health during aging in humans [14], we supposed that the male animals with higher levels of social dominance might display better health. However, according to the reliability test, females performed better with slower aging based on the daily life assessment.

To further validate the reliability of the scale, we estimated half-split reliability by randomly splitting the 12 items into two halves, each comprising six items. Half one included four items on dietary uptake, age and sex, and the remaining six items were in half two. The reliability of half one was 0.831, whereas that of half two was 0.687. From these results, we suggested that dietary uptake performance played a substantially independent role in aging-related health and functioning.

### The validity of the AHFS in rhesus macaques

**Figure 2:**
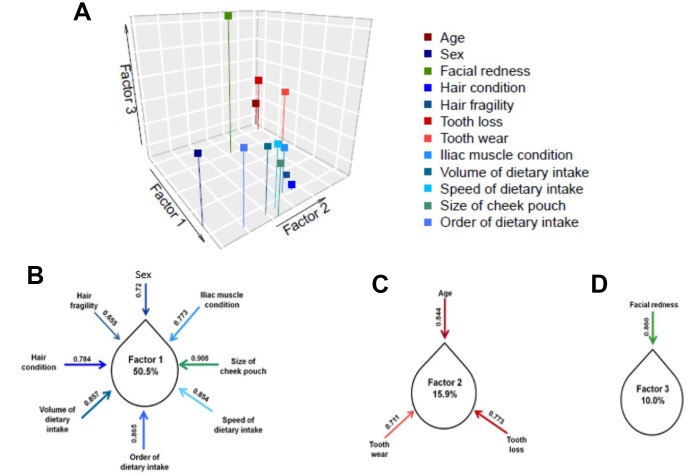
**Using varimax rotation, 12 items on aging-related health and functioning yielded three exploratory factors (Factor 1, Factor 2 and Factor 3).** The factor loading of each item within the explanatory factor is shown in (**B**–**C**). Factor 1, focusing on dietary uptake and physical performance, explained greater than half of the overall variance; Factor 2, focusing on tooth condition, explained 15.9% of the overall variance; and Factor 3, consisting only of perceived age, explained 10% of the overall variance. Notably, each item was considered to significantly contribute to the overall aging-related health and functioning (loading>0.5).

To validate the construction of the scale, we conducted an exploratory factor analysis of the 12 items. The adequacy of the scale was calculated by KMO (0.815), which indicated that these items harbor a dimension-reducible construct. We then divided the 12 items into three distinguishable factors using varimax rotation (Figure 2A). According to the results, three clustered factors together explained 76.4% of the variance in aging-related health and daily functioning. We further estimated the contribution to each factor using the “loading” statistic (Figure 2B–2D). Loadings above 0.5 were considered to contribute to a relatively independent role, helping “denoise” the complexity of aging-related health and functioning. Factor 1 comprised six significantly interrelated items, among which volume of dietary uptake explained the highest level of variance of the 12 items in the AHFS.

### Evaluating the applicability of the AHFS using hierarchical cluster analysis

Hierarchical cluster analysis was conducted to validate the actual applicability of the scale. Individuals’ scores for aging-related health and function were clustered in all subjects as well as within each sex. According to the comparison of the respective presented dendrograms, three clusters were taken, and the optimal number of clusters reflected the severe-aging clustered subgroup, moderate-aging clustered subgroup and mild-aging clustered subgroup. Notably, the average scores of three clustered subgroups displayed significant disparity from each other in all samples, all females and all males (Table 4).

**Table 4:** Three aging-related clusters in different animal subgroups.

**Subgroups based on clustered analysis**	**n**	**Average score**
All animals*		
Severe-aging subgroup	4	29.500 ± 0.577
Moderate-aging subgroup	27	20.259 ± 2.536
Mild-aging subgroup	26	14.231 ± 1.107
Female animals*		
Severe-aging subgroup	4	22.000 ± 1.414
Moderate-aging subgroup	7	17.571 ± 0.787
Mild-aging subgroup	18	14.278 ± 1.074
Male animals*		
Severe-aging subgroup	4	29.500 ± 0.577
Moderate-aging subgroup	18	20.445 ± 1.907
Mild-aging subgroup	6	13.500 ± 0.548

* *P*<0.05 comparing between two clusters

**Figure 3:**
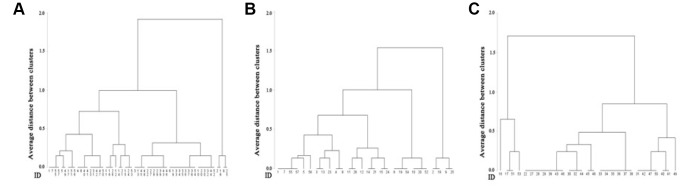
**Depicts three cluster trees based on scores for aging-related health and functioning in all subjects.** (**A**) All females (**B**) and all males (**C**). The distinct patterns of the three trees indicate that the scale can finely separate distinct samples with respect to similarity

The clusters of individual scores in all samples, females and males, were also demonstrated by tree plots (Figure 3). The magnitude of an individual’s score for aging-related health and functioning is represented by the average interval between clusters according to the sequential similarity. In general, the discriminatory capability of the scale validated the applicability of the AHFS as good.

Furthermore, the scores of each item in the three clustered subgroups were also estimated and compared (data not shown). The similar clustered patterns between these composite items and the summed score were revealed, which indicated that the scale could be well separated with respect to the similarity of its composite items.

## DISCUSSION

It is more difficult to quantify aspects of the long and complicated aging-related health and functioning in nonhuman primates than in humans because neither self-rating nor interview procedures are possible in nonhuman animals. We constructed a subjective scale based on daily life observations in healthy captive rhesus macaques. It has been documented that rhesus macaques age at approximately 3 times the rate of humans, with puberty occurring between 2.5 and 4.5 years and a median life span of 27 years [19]. Therefore, we included subjects ranging from young-adult age to young-old age to trace the long and subjective healthy aging process. Due to the complexity of aging, aging health assessments or healthy aging measurements are a continuing challenge in humans. We constructed the present scale based on daily life with the aim to easily and rapidly score captive animals with the least invasiveness. With the present scale, the majority of items could be rated noninvasively, with the exception of tooth condition and muscle palpation in males, which required a very short duration of anesthesia. Furthermore, social traits that nonhuman primates express due to their colony lifestyle could present a sophisticated translational model of aging in humans. For the present scale, candidate items were screened using a step-by-step strategy with the consensus of a multidisciplinary panel. The general three-point scale was believed to easily discriminate the scores for each animal with high intrarater agreement. Therefore, we supposed that the present AHFS could characterize overall health and functioning based on daily life. Notably, accurate measurement of aging-related changes is very difficult. For example, motor functioning can be qualified only using complicated digital-based examination [20]. Therefore, this daily life scale contains the notable merit of practicality. The application of the AHFS could help reduce the bias of baseline variation in animal status to produce higher repeatability and reproducibility in experiments.

In the development of the scale, we selected three domains based on daily life activity: perceived aging, musculoskeletal aging and social aging. Perceived age was reflected by three items. The facial dermis in aged rhesus macaques is thin and fragile [6], which results in the easily recognized darkness of the redness on the face, especially in the periorbital region. Therefore, two experts recommended grading the darkness of facial redness in the rhesus macaques. Intriguingly, we found that perceived facial redness alone explained 10% of the variation in overall aging-related health and functioning. Hair condition, tested in a noninvasive manner, was simultaneously rated. It has been documented that hair condition is closely correlated with immune-endocrine imbalance [21–23], increased social stress [22, 23] and malnutrition [24], all of which can accelerate aging-related decreases in health and functioning. Regarding musculoskeletal aging, we selected the iliac muscle mass as the representative muscle to grade aging-related muscle loss. Although rhesus macaques display a process that matches sarcopenia in humans, a duration of approximately nine years is required to observe 20% muscle loss in elderly macaques [16]. Furthermore, apparent muscle loss is not distinguishable until the animal is >25 years old [16]. Therefore, we used three-point scoring to discriminate the chronic loss of iliac muscle mass. Oral health, as an integral component of overall health, is consistently arousing attention in aging research [25]. It is well known that dental hard tissues do not remodel with aging as bones do. Therefore, we rated tooth loss and tooth wear to reflect the continuous loss of dental hard tissues. Aging-related tooth conditions are also affected by microhabitat determinants, including food, dietary properties and behaviors, in rhesus macaques [26]. Therefore, scoring tooth condition can help integrate the assessment of aging-related health and functioning. The largest aspect of daily life we rated in the scale was social aging, which was reflected in the dietary uptake performance or eating behavior. We supposed that these dietary uptake measures indicated not only nutrition uptake but also essential social activity. Here, we averaged four items, which included the volume of dietary uptake, the speed of eating, the size of the cheek pouch and the order of dietary uptake. Notably, the order of dietary uptake was the only independent social variable that directly assessed the effect of social hierarchy on aging-related health and functioning. Practically, the distance between the interested female and the male was measured to better assess the order of dietary uptake during mealtime. We believe that dietary uptake performance can comprehensively reflect the entangled aging-related health and functioning involving physical function, social status and neuromuscular performance. The term “eating capability” has been quantified for understanding the complicated correlation during dietary management in elderly humans [27]. During dietary uptake, neuromuscular orchestration of forelimb use [28], bimanual coordination [29] and orofacial muscle control [27] can be assessed. Regarding the present scale, both the highest correlation and the greatest loading for an exploratory factor were observed in the four eating-related items. In summary, the present scale that demonstrated high intraobserver reliability, well-explained construct validity and good applicability can help captive rhesus macaques become more valuable in the study of aging-related health and functioning. To the best of our knowledge, no scale has been developed on overall aging-related health and functioning in nonhuman primates. Therefore, no comparison could be carried out between distinct scales.

Of note, the scored aging-related health and functioning in rhesus macaques also displayed some aging-related paradoxes that humans have exhibited. A famous gender-survival paradox during aging has revealed that elderly female humans have worse health and lower functioning but greater longevity than males [30]. During the construction of the present scale, the health of the dominant males within the social hierarchy was not superior to that of females, in contrast to results that high socioeconomic status can retard aging [9, 15], this outcome reveals a gender-related health-social status paradox. Another phenomenon was the calorie-related health-survival paradox during aging. Although a chronic hypercaloric diet has been shown to result in obesity, multisystemic deterioration known as metabolic syndrome and accelerated aging, obese elderly individuals seem to live longer and to have better outcomes against infection compared to their age-matched thinner counterparts [31, 32]. Simultaneously, caloric restriction (CR), which has been found to result in longevity and retarded aging in many lower animals, does not consistently show this effect in nonhuman primates [33]. The largest two rhesus monkey cohorts exposed to a CR intervention initiated in the late 1980s have demonstrated contrasting results between health and survival [19, 34]. In the present rhesus macaque colony, females with lower social rank were observed to have later dietary uptake and smaller cheek pouches, which produced relatively hypocaloric diets and mild CR. However, as the complicated interactions of CR between longer life and better quality of life involved numerous critical co-factors [33, 35–37], whether the mild CR intervention in subjects with worse health and functioning would live longer warrants longitudinal observations. From the perspective of scale utilization, we suggested that these aging-related paradoxes in primates were attributable not only to the complex aging process but also to a lack of dynamic assessment of changeable aspects of health and functioning.

In summary, we developed an observer-based scale by integrating perceived aging, biophysical functioning and socially related performance based on the daily life of rhesus monkeys. The scale has been tested and has shown good interrater reliability, has satisfied construct validity and has acceptable applicability. The limited availability of advanced age samples, especially with healthy status, has hindered us from including adequate samples in the construction of the present scale. We are longitudinally observing and scoring this naturally aging cohort to further supplement the database and improve the scale on aging-related health and functioning. The wide application of the AHFS in rhesus macaques would be very helpful in disentangling the complexity of aging in humans.

## MATERIALS AND METHODS

### Healthy animal screening

We observed all laboratory-maintained group-housed rhesus macaques in the North Primate Research Center affiliated with the Institute of Laboratory Animal Sciences, Chinese Academy of Medical Sciences & Peking Union Medical College from November 2018 to March 2019. Approximately two-thirds of the rhesus monkeys aged 4 to 20 were initially screened as candidates. A total of 57 rhesus monkeys identified as consistently healthy animals with normal functioning were maintained to construct a scale termed the Aging-related Health and Functioning Scale (AHFS). The animals with transient abnormalities, including decreased dietary uptake, diarrhea, fatigue and solitary habits during the observation, were excluded. All selected animals were research-naïve and pregnancy-free during the observation. The sex ratio was 1:1.04 (M:F=28:29). All subjects were of known age, parity, mass, and social rank according to colony records. All protocols were approved by the Institutional Animal Care and Use Committee of the Institute of Laboratory Animal Sciences, Chinese Academy of Medical Sciences & Peking Union Medical College.

### A step-by-step selection of items under panel consensus

A panel including three researchers in the field of human aging, two researchers who study nonhuman primates and five experienced rhesus macaque caretakers was assembled. A step-by-step item selection was performed to screen candidate items for the AHFS. First, the three human aging researchers were consulted to present candidate domains and related items in reference to human aging. Then, items that could be easily differentiated and were relatively noninvasive and occurred in daily life were selected by the two nonhuman primate researchers. Finally, the five rhesus macaque caretakers carried out the preliminary assessment to determine which items could be practically rated. A general 3-point scoring system was used with minor exceptions. The higher the score was, the more severe the age-related aspect of health and functioning.

### Aging-related health and functioning observations and scoring assessment

For the rating of each rhesus monkey, a label designed for the grading week was highlighted. Five experienced caretakers who had undergone training carried out the scale rating as a team. Operationally, two randomly assigned caretakers independently rated each of the candidate animals. If the score disagreed between the two raters, a third caretaker was asked for a rescore. The final score was determined by the agreed upon score among the three raters. If no agreeable score was reached, the score of the animal was determined by consensus.

Perceived aging was graded by facial appearance and hair condition. The darkness of facial redness was subjectively qualified. Hair condition was subjectively graded by glossiness and season-unrelated overall hair loss. Hair glossiness was scored under natural light at a relatively fixed position from a fixed angle. The perceived severity of abnormal hair loss increased the item by one point. Hair fragility was scored by an easy experiment. Three pieces of hair were collected from the spine near the neck. Then, each hair was stuck with glue to a prepared piece of tape with 2 mm extending from the end of the tape. The average force to tear the hair away from the piece of tape was recorded. The extent (easy/moderate/difficult) to which the hair was torn from the piece of tape was used to score hair fragility. Musculoskeletal condition was graded by teeth and representative iliac muscles. The tooth condition was scored by the presence of tooth loss and by the extent of tooth wear. The iliac muscle was assessed by palpating the muscle mass [13] in addition to observing muscle movement. Four items were rated during dietary uptake, including the volume of dietary uptake, speed of dietary uptake per meal, size of the cheek pouch and order of eating the meal. The raters observed three occasions that an animal was eating meals in the group. The dietary uptake performance of each animal was observed for at least three meals, with each observation lasting no less than 10 minutes. The item score was averaged from the three observations.

### Statistical analysis

All observed results on an ordinal scale were analyzed as continuous values. The mean ± standard deviation (SD) was calculated between repetitive assessments. Using the 25^th^ and 75^th^ percentiles as cutoffs, three age groups were defined: junior (8–12 years old), adult (13–16 years old) and senior (17–20 years old). Pearson correlations were used to determine the relationship between the scored AHFS and each of the rated items as well as between pairs of items. In order to examine the reliability of the scale, Cronbach’s alpha value and Cronbach’s alpha half-split value was calculated. In order to validate the construct of the scale, an exploratory factor analysis was performed. The number of underlying exploratory factors in the scale was determined by eigenvalues. Varimax rotation was performed to achieve the simplest structure of these exploratory factors. The factor loadings of each item within its exploratory factor were extracted within the rotated structure. In order to validate the applicability of the scale, a hierarchical cluster analysis was carried out based on the individual’s scored aging-related health and function. The optimal number of clusters was determined based on a comparison of the respective presented dendrograms. Similarity was measured based on the interval of intraindividual scores within each group. All analyses were conducted in SPSS (17.0) and R (3.5.0). A *P* value less than 0.05 was considered statistically significant.
